# Metabolic switching in MASLD: therapeutic roles of ketogenic diet, intermittent fasting, and the gut-liver axis

**DOI:** 10.3389/fnut.2026.1860047

**Published:** 2026-09-04

**Authors:** Purvi Kaur Khanuja, Vrinda Mohan, Alejandro Perez Martinez, Milind Nellutla, Mariam Alamgir, Ayesha Anjum, Fadi Ali Jamaleddin Ahmad, Taiwo Shola Oguntona, Hussam Ahmed Mohamed Ali Saari, Dakshayini Suresh, Nabeel Ansar, Mahmood Alaawad, Zainab Hadi

**Affiliations:** 1 University of North Carolina, Chapel Hill, NC, United States; 2 Department of Medicine, Kasturba Medical College, Manipal, India; 3 Instituto Tecnológico de Estudios Superiores de Monterrey, Guadalajara, Mexico; 4 MNR Medical College & Hospital, Sangareddy, Telangana, India; 5 School of Medicine, St. George's University, True Blue, Grenada; 6 Fatima Memorial Hospital, College of Medicine and Dentistry, Lahore, Pakistan; 7 The University of New Mexico School of Medicine, Albuquerque, NM, United States; 8 Lagos State University College of Medicine, Ikeja, Lagos, Nigeria; 9 Department of Medicine, Privolzhsky Research Medical University, Nizhny Novgorod, Russia; 10 Department of Medicine, University Hospitals Plymouth NHS Trust, Plymouth, United Kingdom; 11 Travancore Medical College And Hospital, Kollam, Kerala, India; 12 Montefiore St Luke’s Cornwall Hospital, Newburgh, NY, United States; 13 College of Medicine, University of Baghdad, Baghdad, Iraq

**Keywords:** gut–liver axis, intermittent fasting, ketogenic diet, metabolic dysfunction-associated steatotic liver disease, metabolic switching, nutritional ketosis, precision nutrition, very-low-energy ketogenic therapy

## Abstract

Metabolic dysfunction-associated steatotic liver disease (MASLD) is common and clinically heterogeneous, with fibrosis stage serving as a major determinant of long-term liver-related risk. This focused narrative review evaluates ketogenic dietary interventions and intermittent fasting as metabolic-switching strategies while explicitly distinguishing conventional or eucaloric ketogenic diets, very-low-energy ketogenic therapy, non-ketogenic low-carbohydrate diets, and distinct fasting protocols. Both approaches can reduce body weight, improve insulin sensitivity, and decrease hepatic steatosis in selected patients, but much of the human evidence is short-term and protocol-dependent. Mechanistic effects attributed to beta-hydroxybutyrate, including inflammasome inhibition, histone modification, and mitochondrial signaling, are supported predominantly by cell and animal studies and have not been directly confirmed in patients with MASLD. The gut-liver axis provides a biologically plausible link through intestinal permeability, microbial metabolites, bile acid signaling, and hepatic innate immunity; however, direct human evidence that ketogenic diets or intermittent fasting restore gut-barrier function remains limited. No trial has directly compared a well-defined ketogenic intervention with documented nutritional ketosis against intermittent fasting. Therefore, relative efficacy, durability, adherence, and weight-loss-independent effects remain uncertain. Clinical use should prioritize fibrosis risk stratification, nutritional adequacy, comorbidity-specific safety, and integration with evidence-based lifestyle and pharmacologic care.

## Highlights

- Ketogenic and intermittent fasting interventions should be classified by protocol, energy intake, and confirmation of nutritional ketosis.
- Short-term human evidence supports improvements in liver fat and metabolic biomarkers, but long-term and fibrosis outcomes remain uncertain.
- Beta-hydroxybutyrate signaling and epigenetic effects are biologically plausible but are supported predominantly by preclinical evidence.
- No direct trial has compared a well-defined ketogenic diet with documented ketosis against intermittent fasting in MASLD.
- Clinical use requires fibrosis risk stratification, a review of nutritional and medication safety, and integration with guideline-based care.

## Introduction

Metabolic dysfunction-associated steatotic liver disease (MASLD), which is frequently diagnosed incidentally, is defined by hepatic steatosis together with at least one cardiometabolic risk factor after consideration of other causes of steatosis and clinically relevant alcohol exposure (1, 2). Formerly referred to as nonalcoholic fatty liver disease (NAFLD), it is now designated as MASLD to better reflect its underlying metabolic drivers without defining the disease simply by the absence of alcohol use (3). MASLD encompasses a spectrum ranging from isolated steatosis to metabolic dysfunction-associated steatohepatitis (MASH), progressive fibrosis, cirrhosis, and hepatocellular carcinoma, with a heterogeneous course rather than an obligatory linear sequence (2, 4, 5). The fibrosis stage is the main determinant of liver-related outcomes, making early risk stratification central to clinical management.

Published prevalence estimates vary. Some reports suggested that more than 38% of people worldwide have MASLD, whereas contemporary pooled analyses estimate that approximately 30–32% of adults worldwide have steatotic liver disease consistent with the former NAFLD definition (6–8). Prevalence is particularly high among people with type 2 diabetes, obesity, central adiposity, dyslipidemia, or hypertension (9). Delayed diagnosis may contribute to serious hepatic and extrahepatic complications. MASLD is an increasingly important cause of cirrhosis, liver transplantation, and hepatocellular carcinoma (HCC) worldwide and is reported to be the leading cause of cirrhosis and liver transplantation in women and the second most common in men (10).

MASLD is more prevalent in men than in women, with approximately 15,731 and 14,310 cases per 100,000, respectively. Peak prevalence has been reported in ages 45–49 years in men and 50–54 years in women. In the United States, approximately 18.5% of men and 10.3% of women have MASLD (11).

Several challenges limit the effectiveness of current lifestyle and pharmacologic interventions for MASLD. Adherence to lifestyle and medication-based therapies can be difficult to sustain; no single treatment is universally effective for all patients, and available therapies differ in effectiveness and safety profiles (12).

Guideline-based management begins with assessment of metabolic comorbidities and fibrosis risk. A simple blood-based score such as the Fibrosis-4 index (FIB-4) is generally used as an initial test, followed by vibration-controlled transient elastography or another second-line method when indicated (2). Lifestyle treatment remains foundational. Clinically meaningful weight reduction improves steatosis and cardiometabolic risk, whereas greater sustained weight loss is generally required to improve steatohepatitis and fibrosis. A Mediterranean-style dietary pattern, reduced intake of ultra-processed foods and sugar-sweetened beverages, regular physical activity, and individualized alcohol counseling form the broader evidence-based lifestyle context (2, 13, 14).

This review has a deliberately narrow focus, examining two approaches that can promote a metabolic shift away from persistent glucose reliance: ketogenic dietary interventions and intermittent fasting (IF). The term ketogenic diet (KD) is used only when carbohydrate restriction is intended to trigger nutritional ketosis. A conventional or eucaloric KD provides adequate energy and generally limits daily carbohydrate intake to approximately 30–50 g/day or less while providing a relatively high proportion of dietary fat. Energy-restricted ketogenic regimens are distinct; very-low-energy ketogenic therapy (VLEKT), formerly termed a very-low-calorie ketogenic diet (VLCKD), typically provides fewer than 800 kcal/day under structured clinical supervision (15, 16). A low-carbohydrate or high-fat diet should not be classified as ketogenic unless ketosis is measured or otherwise documented.

### Literature search strategy and selection criteria

This focused narrative review synthesized literature on KD, VLEKT/VLCKD, non-ketogenic low-carbohydrate diets, intermittent fasting protocols, and the gut-liver axis in MASLD. PubMed and Google Scholar were searched for English-language publications from January 2015 through January 2026, supplemented by major earlier studies and current society guidelines or regulatory documents when relevant. Search terms included “MASLD” or “NAFLD” in combination with “ketogenic diet,” “nutritional ketosis,” “very-low-energy ketogenic therapy,” “low-carbohydrate diet,” “intermittent fasting,” “time-restricted eating,” “alternate-day fasting,” “5:2 diet,” “gut microbiota,” “intestinal permeability,” and “bile acids.”

Human randomized trials, prospective interventions, observational studies, systematic reviews, and meta-analyses were prioritized for clinical outcomes. Preclinical studies were excluded from the assessment of clinical efficacy and were incorporated selectively to describe mechanistic plausibility when relevant human data were lacking. Such claims are explicitly identified as preclinical or experimental. Studies of low-carbohydrate diets without verified ketosis are considered separately from ketogenic interventions and are not used to support ketone-specific signaling claims. The synthesis is qualitative because of substantial heterogeneity in intervention definitions, energy intake, cointerventions, adherence assessment, and outcome measurement.

### Pathophysiology of MASLD

#### Insulin resistance, fatty-acid flux, and lipotoxicity

Hepatic triglyceride accumulation reflects an imbalance among fatty-acid influx, synthesis, oxidation, and secretion. Major sources of hepatic fatty acids include nonesterified fatty acids released from adipose tissue, dietary fatty acids delivered through chylomicron remnants, and hepatic *de novo* lipogenesis from carbohydrate substrates (5, 17–19). Insulin resistance increases adipose lipolysis and hepatic substrate delivery, while compensatory hyperinsulinemia may continue to stimulate sterol regulatory element-binding protein 1c (SREBP1c) and *de novo* lipogenesis. This pattern of selective hepatic insulin resistance helps explain how glucose production and lipogenesis can remain inappropriately active at the same time (5, 18, 19).

Triglyceride storage and lipotoxicity should not be considered interchangeable. Esterification into triglyceride droplets may temporarily buffer excess fatty acids, whereas saturated fatty acids, diacylglycerols, ceramides, free cholesterol, and other harmful lipid species can disrupt insulin signaling, endoplasmic reticulum function, and organelle homeostasis (4, 5, 9). Whether steatosis remains stable or progresses to MASH depends on genetic susceptibility, adipose-tissue dysfunction, diet, physical activity, microbiome-related factors, and the balance between injury and repair.

#### Oxidative stress, mitochondrial dysfunction, and inflammatory signaling pathways

Mitochondrial adaptation is dynamic. Although increased beta-oxidation may initially offset lipid accumulation, sustained substrate excess can increase electron leakage, reactive oxygen species, incomplete oxidation, and mitochondrial stress (20, 21). Oxidative injury activates stress kinases and inflammatory pathways, including c-Jun N-terminal kinase and nuclear factor kappa B. Hepatocytes, Kupffer cells, recruited monocyte-derived macrophages, hepatic stellate cells, endothelial cells, and cholangiocytes contribute to a complex inflammatory and fibrogenic network. Injured hepatocytes release danger signals; macrophage-derived cytokines amplify inflammation; and transforming growth factor beta and related pathways activate stellate cells and extracellular matrix deposition (4, 9, 20–22). Fibrosis therefore reflects the cumulative effects of recurrent injury, immune signaling, and incomplete repair rather than a single cytokine-driven process. Table 1 summarizes the integrated mechanisms and physiological processes underlying MASLD progression.

**Table 1:** Integrated mechanisms and physiological processes underlying MASLD progression.

Process	Principal drivers	Key cells/pathways	Clinical consequence
Fatty-acid influx and *de novo* lipogenesis (5, 17–19)	Adipose insulin resistance, dietary fat, excess carbohydrate, hyperinsulinemia	Adipose lipolysis; chylomicron-remnant delivery; SREBP1c-mediated lipogenesis	Hepatic triglyceride storage; substrate overload; variable progression
Lipotoxicity (4, 5, 9)	Toxic lipid species rather than triglyceride alone	DAG/ceramide signaling, ER stress, membrane and organelle dysfunction	Insulin resistance, hepatocyte stress, inflammatory signaling
Mitochondrial adaptation and dysfunction (20, 21)	Sustained lipid oxidation and nutrient overload	Initially increased oxidation, followed by incomplete oxidation, electron leakage, and ROS	Oxidative injury and impaired metabolic flexibility
Innate and adaptive inflammation (4, 23–27)	Cell injury, LPS, cytokines, danger-associated signals	Kupffer cells, recruited macrophages, lymphocytes, hepatocytes, endothelial cells	MASH activity and amplification of insulin resistance
Fibrogenesis (4, 9)	Repeated injury and incomplete repair	TGF-beta and related signaling activate hepatic stellate cells and matrix deposition	Fibrosis progression, cirrhosis, and liver-related risk

### The gut–liver axis in MASLD

#### Intestinal permeability and endotoxemia

The gut-liver axis is a bidirectional network linking intestinal contents and the liver through portal circulation, bile acids, microbial metabolites, and immune pathways (23–25). The epithelial barrier, mucus layer, tight-junction proteins, antimicrobial peptides, and mucosal immune cells limit hepatic exposure to microbial products. Dysbiosis, increased intestinal permeability, and impaired barrier integrity have been associated with MASLD, although causal direction and interindividual variability remain important sources of uncertainty.

**Figure 1:**
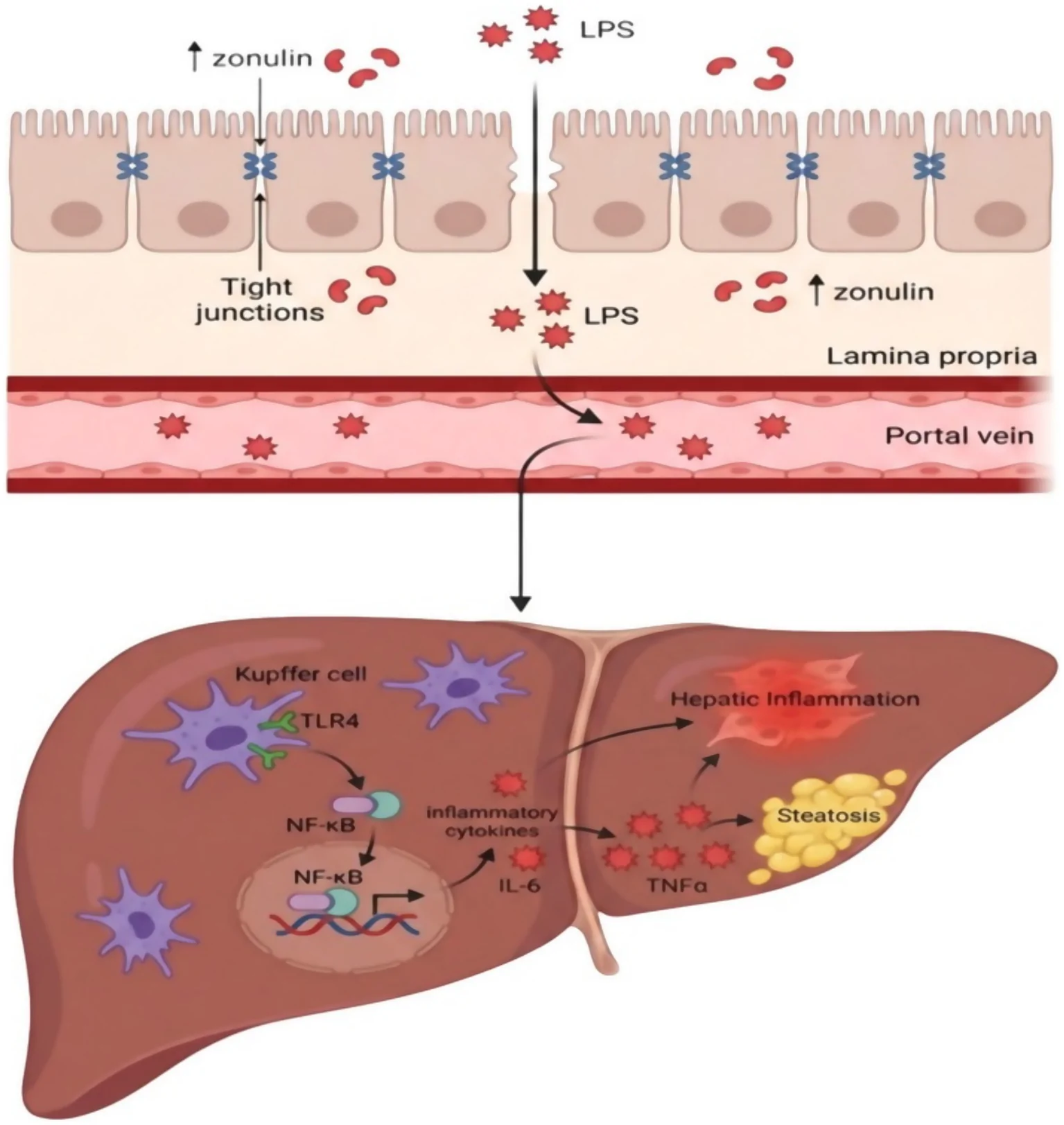
Gut-liver axis in MASLD pathogenesis. Schematic of the bidirectional interaction between the gut and liver in metabolic dysfunction-associated steatotic liver disease (MASLD). Increased intestinal permeability allows lipopolysaccharide (LPS) to translocate into the portal circulation, triggering toll-like receptor 4 (TLR4) signaling and downstream inflammatory pathways, including nuclear factor kappa-light-chain-enhancer of activated B cells (NF-κB). This cascade leads to hepatic inflammation, insulin resistance, and disease progression. LPS, lipopolysaccharide; TLR4, toll-like receptor 4; NF-κB, nuclear factor kappa-light-chain-enhancer of activated B cells; IL-6, interleukin 6; TNF-α, tumor necrosis factor alpha. Created with BioRender.com.

**Figure 2:**
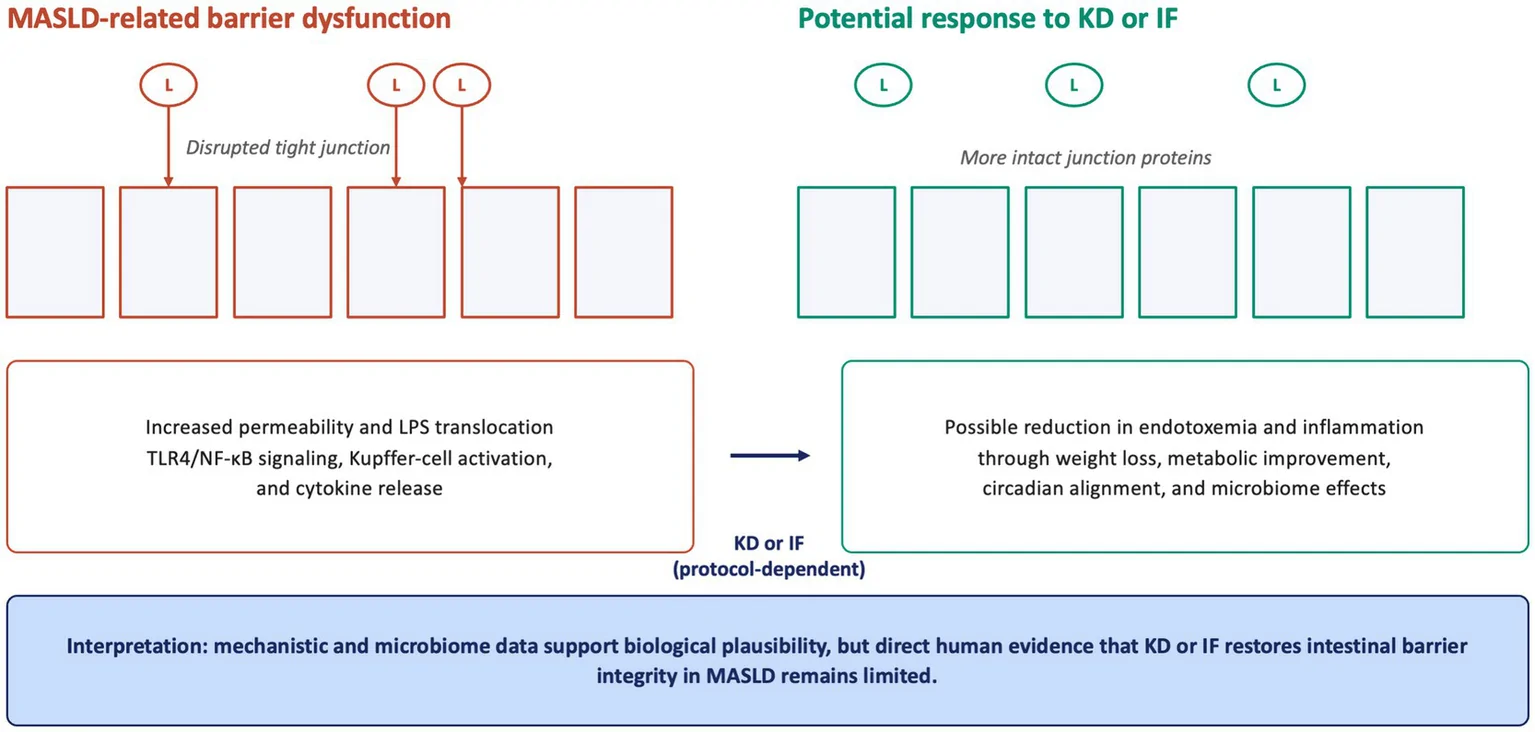
MASLD-related intestinal-barrier dysfunction and the proposed response to ketogenic diets or intermittent fasting. Intervention-focused schematic contrasting MASLD-associated intestinal-barrier dysfunction with a proposed response to ketogenic diets or intermittent fasting. Potential changes in permeability, microbial metabolism, portal LPS exposure, and hepatic inflammatory signaling are presented as protocol-dependent mechanisms. Direct human evidence that KD or IF restores gut-barrier function in MASLD remains limited. IF, intermittent fasting; KD, ketogenic diet; LPS, lipopolysaccharide; MASLD, metabolic dysfunction-associated steatotic liver disease; NF-κB, nuclear factor kappa B; TLR4, toll-like receptor 4.

When barrier function is impaired, pathogen-associated molecular patterns such as lipopolysaccharide (LPS) can enter the portal circulation. LPS activates toll-like receptor 4 signaling in Kupffer cells and other hepatic cell types, leading to nuclear factor kappa B activation and release of tumor necrosis factor alpha, interleukin-6, and other mediators (23–27). This pathway may worsen insulin resistance, inflammation, and fibrogenic signaling. Figure 1 depicts this disease-pathogenesis cascade and is intentionally distinct from Figure 2, which focuses on the proposed effects of dietary interventions and the limitations of the supporting evidence.

#### Bile acids and microbial metabolites

Gut microbiota also convert primary bile acids into secondary bile acids and modulate farnesoid X receptor (FXR) and Takeda G-protein-coupled receptor 5 (TGR5) signaling. These pathways influence lipid and glucose metabolism, intestinal integrity, and immune regulation (25, 28). Short-chain fatty acids, indole derivatives, endogenous ethanol, and other microbial metabolites may also be relevant; however, microbial signatures vary among populations and are not currently suitable for selecting KD or IF in routine care.

### Metabolic switching as a therapeutic target

Ketogenesis is the hepatic production of acetoacetate, acetone, and beta-hydroxybutyrate (BHB) when carbohydrate availability and insulin signaling are sufficiently reduced. Ketosis is the physiological state in which circulating ketone bodies become a meaningful fuel source. Fasting, prolonged exercise, and a well-formulated KD can induce this state (29–31). In contrast, a low-carbohydrate diet may improve energy balance or glycemia without producing sustained nutritional ketosis.

**Figure 3:**
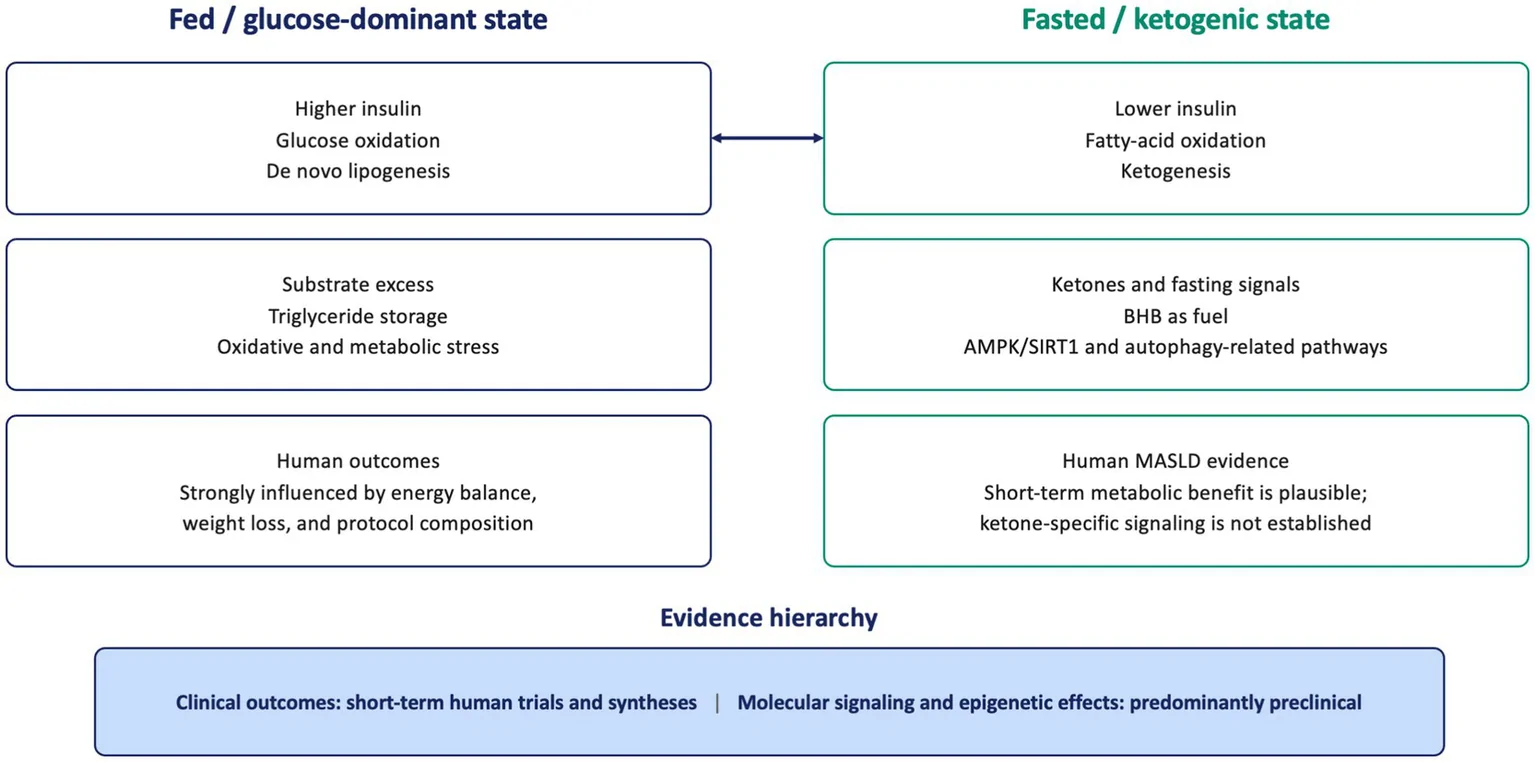
Glucose-dominant and fasted/ketogenic metabolic states with an evidence hierarchy. Schematic comparing glucose-dominant and fasted/ketogenic metabolic states. The figure separates clinically observed short-term outcomes, including changes in weight, liver fat, insulin sensitivity, and selected liver enzymes, from ketone-specific molecular mechanisms that remain predominantly preclinical. AcAc, acetoacetate; AMPK, AMP-activated protein kinase; BHB, beta-hydroxybutyrate; MASLD, metabolic dysfunction-associated steatotic liver disease; SIRT1, sirtuin 1.

Ketone bodies have functions beyond serving as energy substrates. Experimental studies show that BHB can regulate G protein-coupled receptors, suppress the NLRP3 inflammasome, influence histone deacetylation and lysine beta-hydroxybutyrylation, and modify oxidative stress responses (29, 30, 32). Evidence for these signaling and epigenetic effects comes primarily from cellular systems and animal models. Although these findings provide biological plausibility, their magnitude and clinical relevance have not been directly established in people with MASLD. Figure 3, therefore, distinguishes clinically observed human outcomes from predominantly preclinical molecular mechanisms.

Clinical studies and meta-analyses indicate that intermittent fasting (IF) may reduce body weight and improve insulin sensitivity, aminotransferase levels, and hepatic fat measures over several weeks to months; however, outcomes vary by protocol and frequency and are often associated with caloric restriction and weight loss (1, 33–36). Likewise, carbohydrate-restricted interventions can reduce hepatic fat and improve glycemic or lipid measures (11, 37–44).

### Gut-barrier reprogramming and hepatic inflammation

Diet quality influences intestinal barrier biology. Mediterranean-style dietary patterns are generally associated with greater fiber and polyphenol intake, whereas Western dietary patterns rich in ultra-processed foods may promote dysbiosis and impaired barrier integrity (13, 14). These observations provide relevant background but do not demonstrate that KD or IF necessarily produces the same effects.

Human evidence that a ketogenic diet restores intestinal tight junction integrity in MASLD is limited. Ketogenic interventions may influence Akkermansia, bile-salt hydrolase activity, short-chain fatty acid producers, and bile-acid metabolism, but findings vary based on fat quality, fiber intake, energy deficit, medication exposure, and baseline microbiome composition (27, 45). IF may influence microbial rhythmicity, short-chain fatty-acid production, and endotoxemia through meal timing and energy restriction (46–48). Most available data come from small cohorts, populations without MASLD, or secondary mechanistic literature. Zonulin is also an imperfect, assay-dependent marker of permeability and should not be considered a definitive measure of barrier restoration.

Figure 2, therefore, presents barrier improvement as a proposed, protocol-specific mechanism rather than a confirmed clinical outcome. Potential benefits may result from weight loss, improved glycemic control, reduced hepatic lipid accumulation, circadian alignment, or altered microbial metabolism. The extent to which these changes independently reduce portal LPS exposure or Kupffer-cell activation in people with MASLD remains uncertain.

### Ketogenic diets and intermittent fasting: evidence-informed comparison

KD and IF share several downstream effects, including lower average insulin exposure, greater reliance on fatty acid oxidation, and potential activation of AMPK, SIRT1, and autophagy-related pathways, but they achieve these effects through different nutritional and behavioral structures (46, 49–51). A conventional ketogenic diet changes daily macronutrient intake, whereas intermittent fasting changes the timing or frequency of food intake and may not reduce total caloric intake. VLEKT combines ketosis with severe energy restriction, a formula-based structure, and close clinical supervision; therefore, its outcomes cannot be attributed to ketosis alone (15, 16).

Clinical evidence is strongest for short-term changes in weight, liver fat, insulin sensitivity, and aminotransferases. Evidence is substantially weaker for fibrosis regression, hard clinical outcomes, long-term nutritional adequacy, and benefits independent of weight loss. The randomized comparison by Holmer et al. evaluated a low-carbohydrate, high-fat diet against 5:2 intermittent calorie restriction (52). Because nutritional ketosis was not documented, this study is not a direct KD-versus-IF comparison and cannot establish the comparative effectiveness of ketone-mediated treatment.

No existing trial has directly compared a well-defined ketogenic intervention with documented nutritional ketosis against a standardized intermittent fasting (IF) protocol in people with MASLD. Accordingly, claims that IF is inherently more sustainable or that ketogenic diets (KD) produce greater short-term effects are not supported by direct comparative evidence. In practice, selection should be individualized according to patient preferences, fibrosis risk, diabetes management, renal and hepatic function, food availability, cultural considerations, history of disordered eating, and ability to adhere to monitoring (15, 16, 46, 49–52). Table 2 summarizes the evidence without implying superiority.

**Table 2:** KD vs. IF.

Domain	Ketogenic interventions	Intermittent fasting
Definition	Conventional/eucaloric KD: severe carbohydrate restriction intended to induce ketosis with adequate energy. VLEKT/VLCKD: usually <800 kcal/day and medically supervised. Non-ketogenic LCD/LCHF should be analyzed separately (15, 16, 76).	TRE, alternate-day fasting, periodic fasting, or 5:2 energy restriction. Ramadan fasting is a cultural/religious exposure rather than a prescribed clinical protocol (35, 46, 50, 77).
Best-supported human outcomes	Short-term reductions in weight, liver fat, and insulin resistance; much clinical evidence derives from energy-restricted programs or unverified ketosis (41–44).	Short-term reductions in weight, liver fat, and insulin resistance; magnitude varies by protocol and energy deficit (1, 34, 58, 61, 78, 79).
Mechanistic evidence	Fatty-acid oxidation and ketogenesis are established physiology; BHB inflammasome, epigenetic, and direct antifibrotic effects are mainly preclinical (29, 30, 32, 45, 49).	Meal timing, energy restriction, circadian alignment, and autophagy are plausible; direct human hepatic autophagy and gut-barrier evidence is limited (46, 47, 50, 51, 80, 81).
Key confounders	Energy restriction, weight loss, protein adequacy, fat quality, and whether ketosis was actually achieved (15, 16, 42, 52).	Total energy intake, compensatory eating, fasting-window adherence, meal timing, and medication adjustment (50, 52, 57).
Safety considerations	Ketogenic symptoms, constipation, variable dyslipidemic response, micronutrient/fiber adequacy, SGLT2 inhibitor-associated ketoacidosis, and advanced liver/kidney disease (14, 16, 45, 82).	Hypoglycemia with glucose-lowering therapy, dizziness, fatigue, headache, disordered-eating risk, frailty, and pregnancy (14, 50, 82, 83).
Comparative certainty	No direct trial versus standardized IF with documented nutritional ketosis (52).	The LCHF-versus-5:2 trial is informative but is not a true KD-versus-IF comparison because ketosis was not documented (52).
Clinical interpretation	Potential option when carefully defined, nutritionally adequate, monitored, and aligned with patient preference (2, 14, 82, 83).	Potential option when the schedule is safe, feasible, and sustainable for the individual patient (2, 14, 50, 83).

### Clinical translation

#### Patient selection and monitoring

Potential candidates for a restrictive dietary regimen include adults with noncirrhotic MASLD, obesity or central adiposity, insulin resistance, and a strong preference for the selected protocol. Before initiation, clinicians should assess fibrosis risk, nutritional status, sarcopenia or frailty, renal function, glycemic management, cardiovascular risk, pregnancy status, and history of disordered eating. Advanced cirrhosis, decompensation, inadequate nutritional reserves, pregnancy or lactation, childhood, and significant eating disorders generally warrant avoidance or specialist-directed care. Patients taking insulin, sulfonylureas, or sodium-glucose cotransporter 2 inhibitors require medication review because fasting or carbohydrate restriction may increase the risk of hypoglycemia or euglycemic ketoacidosis.

**Figure 4:**
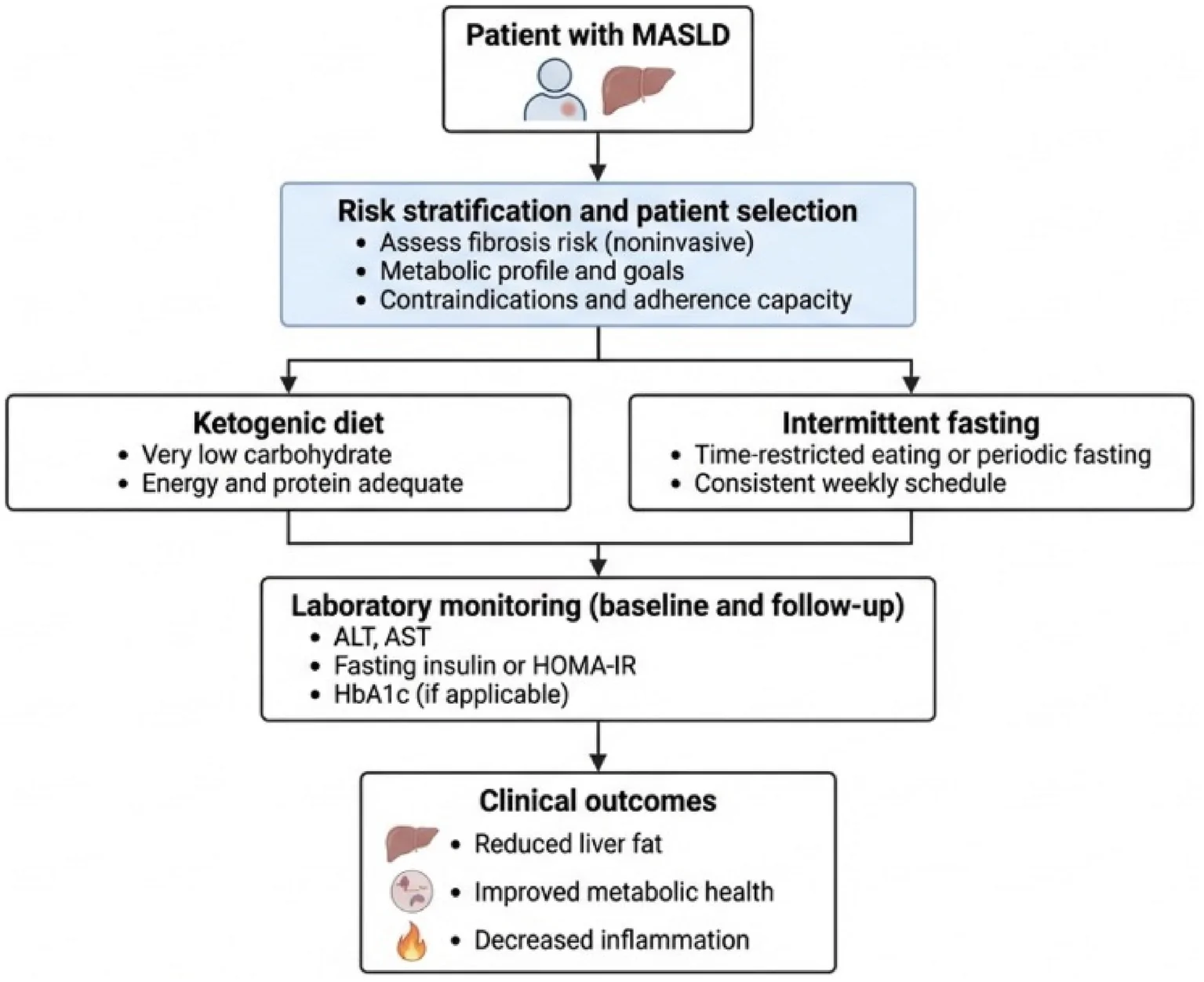
Clinical translation of metabolic dietary interventions in MASLD. Proposed framework for clinical implementation of ketogenic diets (KD) and intermittent fasting (IF) in MASLD, including fibrosis-risk assessment, patient selection, metabolic and hepatic monitoring, and individualized evaluation of outcomes. ALT, alanine aminotransferase; AST, aspartate aminotransferase; HbA1c, hemoglobin A1c; HOMA-IR, homeostatic model assessment of insulin resistance; IF, intermittent fasting; KD, ketogenic diet; MASLD, metabolic dysfunction-associated steatotic liver disease. Created with BioRender.com.

Monitoring intensity should match the intensity of intervention. Baseline and follow-up assessments may include weight and waist circumference, blood pressure, dietary intake, liver enzymes, renal function, electrolytes, glycemia, hemoglobin A1c, lipid profile, and symptoms. When a ketogenic diet is prescribed, measured blood BHB can confirm attainment of ketosis and improve interpretability. FIB-4 and secondary elastography should be used for fibrosis-risk assessment rather than as short-term dietary-response markers (2). A dietitian-led, multidisciplinary approach can support protein, micronutrient, and fiber adequacy while reducing attrition. Figure 4 summarizes key elements for the clinical implementation of metabolic dietary interventions in MASLD.

#### Pharmacotherapy as an adjunct to lifestyle treatment

Dietary strategies should complement rather than replace disease-modifying therapy. Resmetirom received U.S. Food and Drug Administration approval in 2024 for adults with noncirrhotic MASH/NASH and moderate-to-advanced fibrosis used with diet and exercise (53, 54). In 2025, semaglutide, a GLP-1 agonist, received an indication for adults with MASH and moderate-to-advanced fibrosis for use in conjunction with diet and exercise, based on interim histologic results from the ESSENCE program (55, 56). Pharmacotherapy remains phenotype- and label-specific; dietary interventions should be coordinated with treatment of obesity, diabetes, hyperlipidemia, and hypertension.

#### Monitoring and safety

Available short-term evidence suggests that IF and KD can be implemented without major safety signals in selected patients with metabolic liver disease; clinical studies have generally not shown clinically important adverse changes in lipid profiles, liver enzymes, glycemic markers, blood pressure, or body composition during the intervention period (45). Studies of IF and low-carbohydrate regimens also emphasize the need for structured monitoring rather than routine exclusion of these dietary approaches. Baseline evaluation should include an assessment of hepatic steatosis and fibrosis risk, liver and renal function, metabolic markers, and nutritional status, with follow-up of liver enzymes, weight, and metabolic parameters to assess efficacy and identify adverse effects (57).

Although serious adverse events appear uncommon, mild symptoms such as dizziness, fatigue, or gastrointestinal intolerance have been reported, supporting individualized follow-up and multidisciplinary oversight (58). Figure 4 provides a clinical framework for risk-stratification, patient selection, monitoring, and outcome assessment in MASLD.

#### Biomarker monitoring

Biochemical and imaging assessments should be interpreted according to their intended clinical role. Aminotransferases and metabolic markers can change over weeks to months, whereas FIB-4 and the NAFLD Fibrosis Score (NFS) are fibrosis-risk stratification tools and should not be treated as validated markers of short-term responses to KD or IF. Imaging-based liver-fat measurements are more sensitive for detecting changes in steatosis, while elastography provides complementary information about fibrosis risk. Table 3 summarizes commonly used monitoring measures and their interpretation during these interventions.

**Table 3:** Monitoring measures in MASLD and their interpretation during KD or IF.

Measure	Clinical role	Interpretation during intervention	References
ALT/AST	Hepatocellular injury; may be normal despite advanced disease	Can decrease with weight loss and liver-fat reduction; not a fibrosis measure	(43, 44, 79)
GGT	Oxidative/metabolic liver stress; nonspecific	Short-term decreases reported; VLEKT and energy restriction must be distinguished	(42, 84)
HOMA-IR/fasting insulin	Insulin resistance	Often improves with weight loss, carbohydrate restriction, or fasting	(80, 85)
Lipid profile	Cardiometabolic risk and intervention safety	Triglycerides often fall; LDL response to KD is variable and requires monitoring	(86, 87)
CRP and cytokines	Systemic inflammation; limited specificity	May improve with weight loss; BHB-specific anti-inflammatory effects in humans remain uncertain	(81, 88)
Zonulin/LPS-related measures	Research markers of permeability/endotoxemia	Assay limitations and limited human MASLD validation; not routine clinical markers	(48, 89)
FIB-4	First-line fibrosis-risk score based on age, AST, ALT, platelet count	Use for baseline risk stratification and longitudinal clinical context; it is not a validated short-term diet-response biomarker	(2)
NFS	Composite fibrosis-risk score including age, BMI, glycemia, platelets, albumin, AST/ALT ratio	May complement risk assessment; performance varies and is not specific to diet response	(2)
MRI-PDFF/CAP	Quantitative or semiquantitative liver-fat assessment	Preferred objective outcomes for steatosis change when available	(61)
Transient elastography	Liver stiffness and fibrosis risk	Interpret with inflammation and weight change; useful as second-line assessment after FIB-4	(2)

## Discussion: integrated synthesis and clinical position

Current evidence suggests that ketogenic dietary interventions and intermittent fasting can improve short-term metabolic and hepatic surrogate outcomes when they produce meaningful and sustained changes in energy balance, meal timing, or carbohydrate intake. Evidence is more consistent for reductions in liver fat and improvements in insulin sensitivity than for fibrosis. Diminishing effects over time may reflect reduced adherence, metabolic adaptation, compensatory intake, loss of dietary structure, or weight regain; however, long-term comparative data are limited. The intervention label alone is therefore insufficient to guide selection. A supervised very low-energy ketogenic diet may produce rapid weight loss in select patients but requires a different level of monitoring and nutritional restriction than an eucaloric ketogenic diet. Time-restricted eating may be practical for some patients but challenging for shift workers, people taking glucose-lowering medications, or those unable to maintain a regular schedule. Alternate-day or 5:2 energy restrictions may reduce weekly caloric intake but can also lead to hunger, fatigue, and compensatory eating. The preferred clinical option is one that is safe, nutritionally adequate, compatible with the patient’s lifestyle, and demonstrably effective at follow-up.

**Figure 5:**
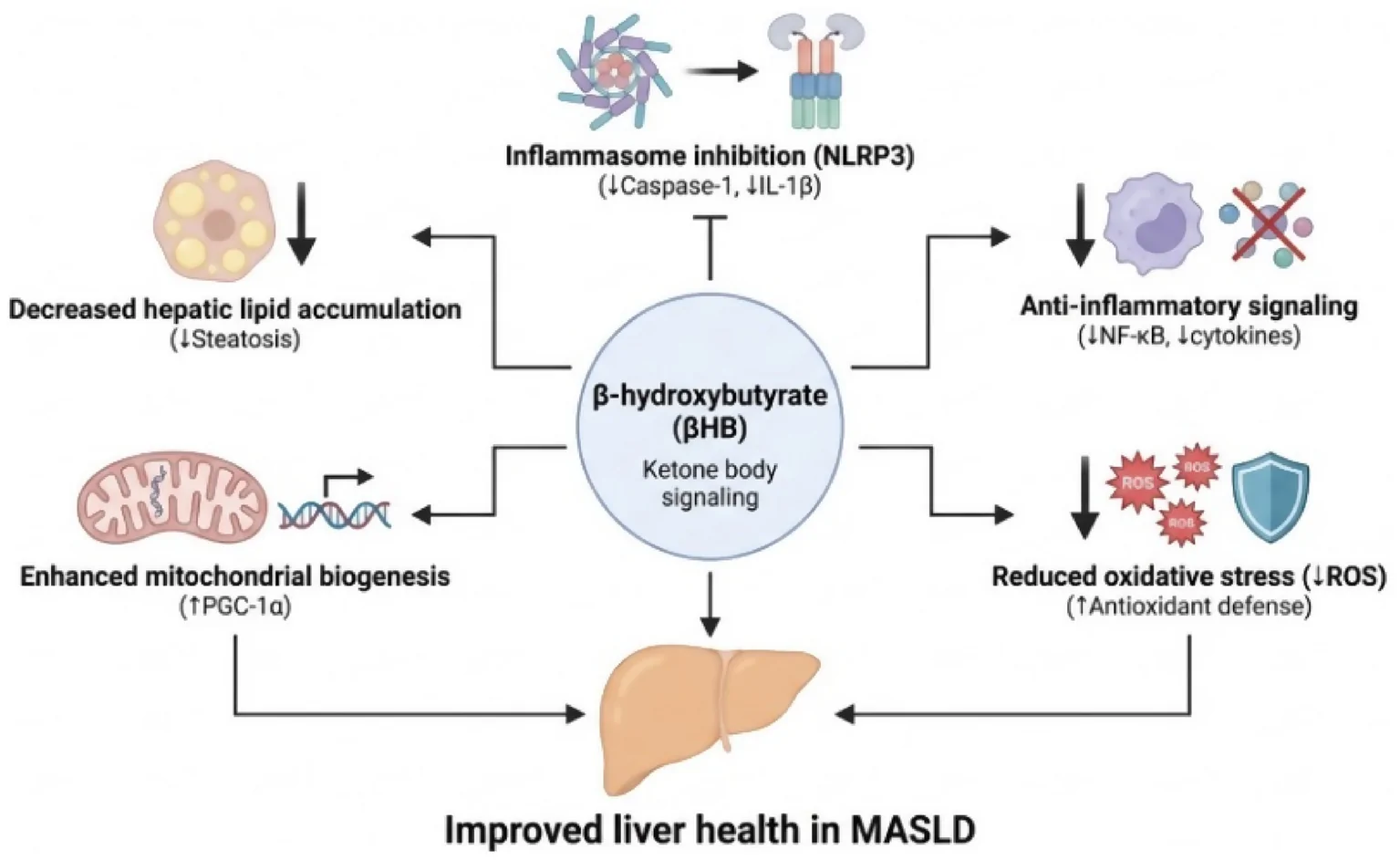
Proposed ketone-body signaling pathways in MASLD. Hypothesis-generating schematic of proposed BHB-mediated metabolic and signaling pathways, including inflammasome, oxidative stress, mitochondrial, and inflammatory effects. Many ketone-specific molecular effects shown are supported predominantly by preclinical evidence and should not be interpreted as established clinical outcomes in MASLD. BHB, beta-hydroxybutyrate; NLRP3, NOD-like receptor family pyrin domain-containing 3; NF-κB, Nuclear factor kappa B; ROS, reactive oxygen species; PGC-1α, Peroxisome proliferator-activated receptor gamma coactivator 1-alpha. Created with BioRender.com.

**Figure 6:**
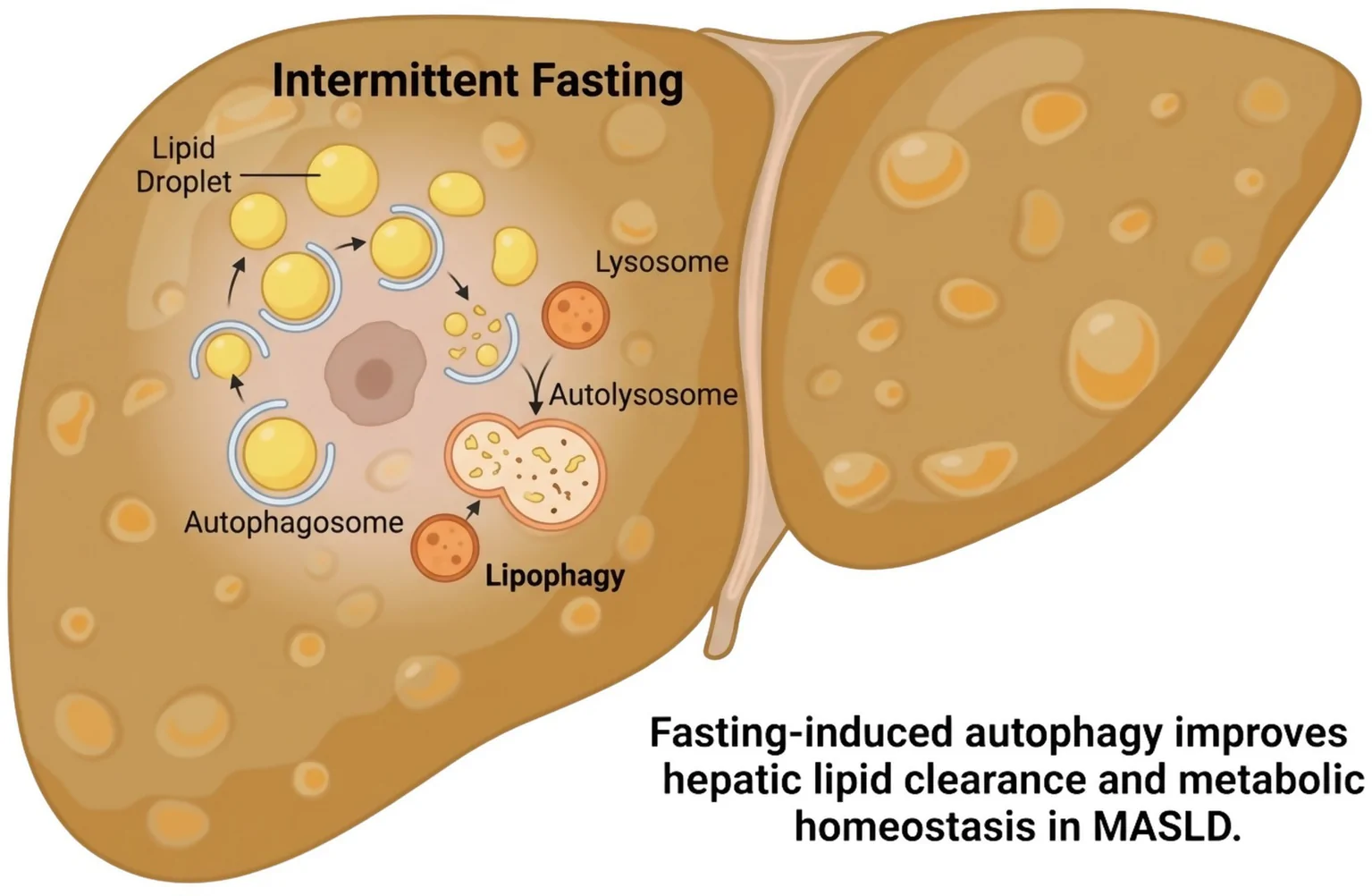
Fasting-induced autophagy and lipophagy in MASLD. Conceptual schematic illustrating fasting-associated autophagy and lipid-droplet clearance. The pathway is mechanistically plausible, but direct evidence that these hepatic processes mediate clinical benefit in human MASLD remains limited. IF, intermittent fasting; MASLD, metabolic dysfunction-associated steatotic liver disease. Created with BioRender.com.

The evidence can be conceptualized in three tiers. First, short-term human studies support improvements in weight, liver fat, glycemic control, and selected liver enzymes. Second, mechanistic evidence in humans involving the microbiome, bile acids, autophagy, and ketone-signaling pathways is emerging but inconsistent. Third, many specific molecular claims, particularly those involving BHB-mediated epigenetic modulation and direct antifibrotic signaling, remain predominantly preclinical. Maintaining these distinctions helps prevent mechanistic plausibility from being presented as established clinical benefit. Figure 5 is a hypothesis-generating model of proposed ketone-body signaling pathways. Many BHB-mediated inflammatory, antioxidant, and mitochondrial effects shown in the figure are supported mainly by preclinical studies and should not be interpreted as proven clinical outcomes. Figure 6 provides a conceptual illustration of autophagosome formation and lipid-droplet clearance during fasting; direct evidence for these hepatic pathways in human MASLD remains limited.

### Knowledge gaps and future directions

#### Need for long-term randomized trials and observational studies

Although randomized clinical trials of KD- and IF-related interventions have reported improvements in hepatic steatosis, insulin sensitivity, and metabolic biomarkers (37, 59, 60), evidence on long-term efficacy, sustainability, and safety remains limited. In particular, the durability of hepatic fat reduction, cardiovascular outcomes, and effects on fibrosis progression are insufficiently characterized (61–63). Large, adequately powered, long-term randomized trials and multicenter cohort studies with sufficient follow-up are needed to assess adherence, nutritional adequacy, adverse effects, and comparative outcomes across dietary and lifestyle strategies (27, 64, 65). Additional research is also needed in lean or low-body mass index (BMI) populations, including Asian populations and individuals with lower BMI (66).

#### Biomarkers of response

A major unresolved issue is the identification of reliable and reproducible biomarkers for monitoring response to IF and KDs in MASLD (67). Heterogeneity in metabolic phenotype, gut microbiota (68), and genetic susceptibility likely contributes to variable clinical outcomes. Candidate measures include circulating ketone concentrations, indices of insulin resistance, inflammatory biomarkers, and bile-acid (BA) profiles. Integrated approaches using metabolomics, lipidomics, and microbiome profiling may help distinguish responders from nonresponders and support more precise dietary interventions (69).

#### Future directions

Despite growing evidence supporting KD and IF in MASLD, several gaps warrant further investigation. First, large-scale RCTs directly comparing KD with IF are lacking. Existing studies are generally short-term and heterogeneous in their dietary protocols, limiting conclusions about sustained efficacy, safety, and comparative effectiveness across diverse MASLD populations. Second, the gut microbiome is an important area for further study in MASLD, including its role in pathogenesis and treatment response. Prebiotics, probiotics, synbiotics, and postbiotics could be investigated as adjuncts to KD and IF. Modulation of microbial composition and function may enhance gut barrier integrity, reduce endotoxemia, and improve metabolic outcomes, but these approaches require further clinical validation (70, 71).

Third, combining dietary strategies with pharmacologic therapies, particularly incretin-based agents such as glucagon-like peptide-1 (GLP-1) receptor agonists, is another area for investigation. GLP-1-based therapies can support weight reduction and insulin sensitivity and may influence hepatic lipid synthesis, with potential synergy with metabolic switching induced by KD or IF that warrants further study (72, 73).

**Figure 7:**
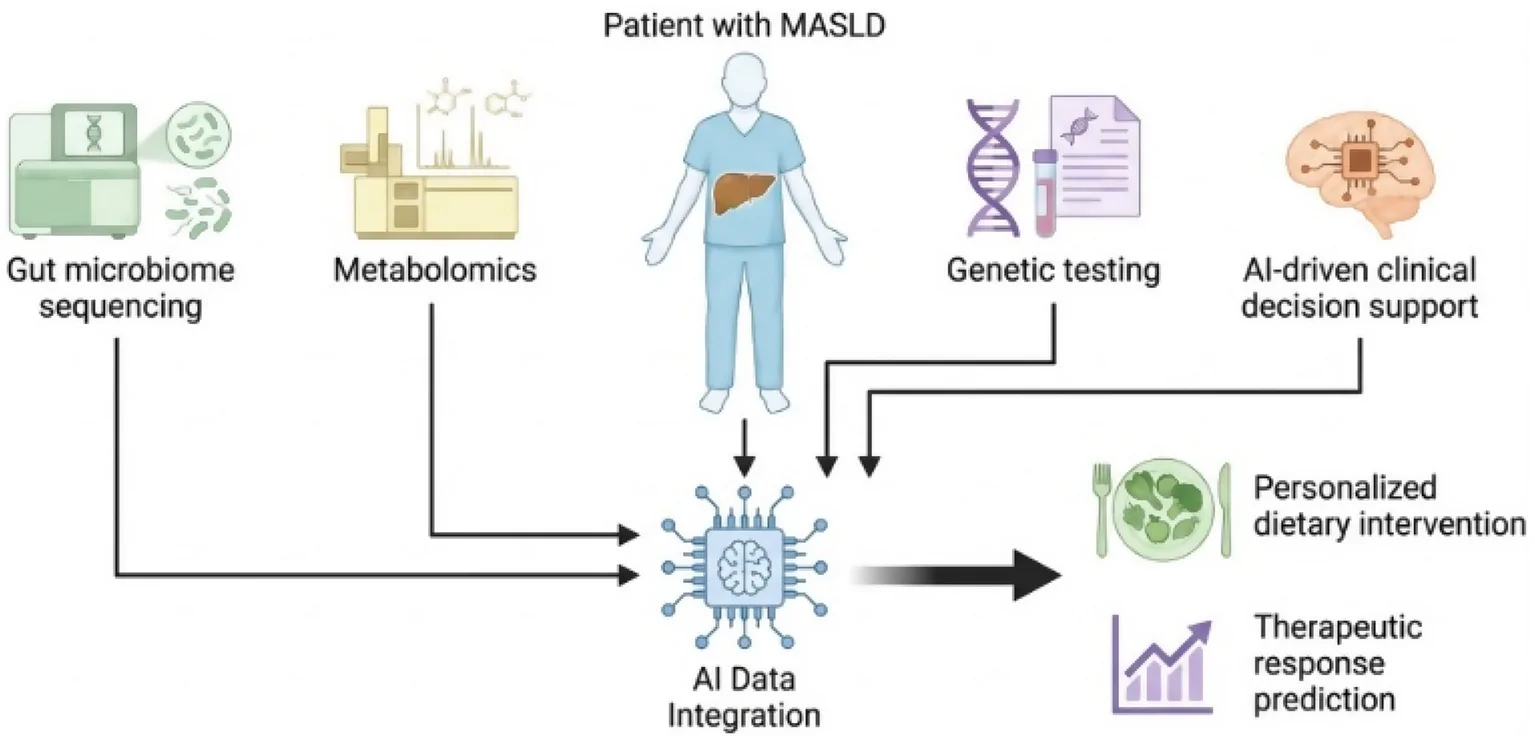
Precision nutrition in MASLD. Conceptual model integrating clinical phenotype, metabolic profiling, gut-microbiome analysis, biomarkers, genetics, and AI-supported decision tools with dietary strategies such as KD and IF. These approaches require prospective validation and should supplement, not replace, established fibrosis-risk assessment and clinical judgment. AI, artificial intelligence; IF, intermittent fasting; KD, ketogenic diet; MASLD, metabolic dysfunction-associated steatotic liver disease. Created with BioRender.com.

Fourth, precision nutrition offers a potential framework for individualizing dietary therapy in MASLD. Validation of biomarkers, including circulating metabolites; inflammatory markers, gut-derived signals such as zonulin and LPS; and genetic or epigenetic signatures, could help clinicians tailor KD or IF approaches to patient-specific metabolic profiles and predicted response. A personalized strategy that integrates metabolic features, gut microbiota, and lifestyle factors may improve therapeutic outcomes and adherence compared with a uniform approach. Figure 7 presents a proposed integration of clinical and molecular data, including artificial intelligence (AI)-supported decision tools, for precision nutrition in MASLD.

Finally, multi-omics technologies, including metabolomics, metagenomics, transcriptomics, and proteomics, may help clarify the complex interactions underlying MASLD and support the development of more targeted, individualized therapeutic strategies (74, 75).

## Study limitations

This narrative review has several limitations, including selection bias, restriction to English-language studies, and heterogeneity in intervention terminology and outcome reporting. Although preclinical studies were excluded from the clinical-effect analysis, experimental and secondary mechanistic literature was selectively cited to illustrate biological pathways; many claims involving BHB signaling, epigenetics, autophagy, and gut-barrier function have not yet been directly verified in patients with MASLD. Several studies labeled as ketogenic did not confirm ketosis, and many clinical findings derive from VLEKT/VLCKD or non-ketogenic low-carb interventions. No trial directly compares a well-defined ketogenic diet with verified nutritional ketosis against a standardized intermittent fasting protocol. Most available studies are short-term, small, and confounded by factors such as weight loss, energy restriction, and intensive behavioral support. Consequently, long-term adherence, nutritional adequacy, cardiovascular outcomes, fibrosis progression, and effects independent of weight loss remain uncertain.

## Conclusion

Ketogenic diets and intermittent fasting are promising adjunctive strategies for adults with MASLD, but their precise roles remain uncertain. Human evidence supports potential short-term reductions in liver fat and improvements in metabolic risk, whereas proposed mechanisms involving ketone signaling, epigenetic regulation, gut-barrier restoration, and antifibrotic effects remain largely mechanistic or preclinical. Conventional ketogenic diets, VLEKT/VLCKD, non-ketogenic low-carbohydrate diets, and distinct fasting protocols should not be treated as interchangeable interventions. In the absence of direct comparative trials, their relative effectiveness, sustainability, and benefits independent of weight loss remain unclear. Clinical use should be individualized, monitored for efficacy and safety, designed to preserve nutritional adequacy, and integrated with fibrosis-risk assessment, guideline-based lifestyle management, and appropriate pharmacotherapy.
